# Whole-Genome Sequencing of Rice Mutant Library Members Induced by N-Methyl-N-Nitrosourea Mutagenesis of Fertilized Egg Cells

**DOI:** 10.1186/s12284-022-00585-1

**Published:** 2022-07-16

**Authors:** Takahiko Kubo, Yoshiyuki Yamagata, Hiroaki Matsusaka, Atsushi Toyoda, Yutaka Sato, Toshihiro Kumamaru

**Affiliations:** 1 Faculty of Agriculture, Kyushu University, 744 Motooka, Nishi-ku, Fukuoka 819-0395 Japan; 2 National Institute of Genetics, Yata 1111, Mishima, Shizuoka 411-8540 Japan

**Keywords:** Rice mutant library, *N*-methyl-*N*-nitrosourea (MNU), Single nucletide variant (SNV), NGS, In silico TILLING, *Oryza sativa*, Whole-genome sequencing, Genetic resource

## Abstract

Although targeted genome editing technology has become a powerful reverse genetic approach for accelerating functional genomics, conventional mutant libraries induced by chemical mutagens remain valuable for plant studies. Plants containing chemically induced mutations are simple yet effective genetic tools that can be grown without regard for biosafety issues. Whole-genome sequencing of mutant individuals reduces the effort required for mutant screening, thereby increasing their utility. In this study, we sequenced members of a mutant library of *Oryza sativa* cv. Nipponbare derived from treating single fertilized egg cells with *N*-methyl-*N*-nitrosourea (MNU). By whole-genome sequencing 266 M_1_ plants in this mutant library, we identified a total of 0.66 million induced point mutations. This result represented one mutation in every 146-kb of genome sequence in the 373 Mb assembled rice genome. These point mutations were uniformly distributed throughout the rice genome, and over 70,000 point mutations were located within coding sequences. Although this mutant library was a small population, nonsynonymous mutations were found in nearly 61% of all annotated rice genes, and 8.6% (3248 genes) had point mutations with large effects on gene function, such as gaining a stop codon or losing a start codon. WGS showed MNU-mutagenesis using rice fertilized egg cells induces mutations efficiently and is suitable for constructing mutant libraries for an in silico mutant screening system. Expanding this mutant library and its database will provide a useful in silico screening tool that facilitates functional genomics studies with a special emphasis on rice.

**Supplementary Information:**

The online version contains supplementary material available at 10.1186/s12284-022-00585-1.

## Background

Nearly a century has passed since two geneticists, Muller (1927) and Stadler (1928), first reported that X-rays could induce mutations and increase the mutation rate compared with spontaneous mutations. After this landmark discovery, additional mutagens have been discovered and used in plant breeding and plant genetic research. As of 2021, 3365 cultivars (229 plant species) originating from artificially produced mutants have been developed and released (the Joint FAO/IAEA mutant database administered by the Food and Agriculture Organization/International Atomic Energy Agency, 2021). Of the 3365 cultivars, 25.3% (853 cultivars) are rice (*Oryza sativa* L.) mutants. This fact indicates that artificial mutants are good sources of genetic variation for cultivar improvement, especially in rice.

Chemical mutagens are relatively easy to use agents. Alkylating agents such as ethyl methanesulfonate (EMS) and N-methyl-N-nitrosourea (MNU) are frequently used to induce mutations in plants. Alkylating agents alkylate guanine nucleobases, resulting in G/C to A/T transitions irrespective of the genomic region. Mature, dry seed is most commonly used in chemical mutagenesis treatments because alkylating agents are toxic for growing plants. Satoh and Omura (1979) developed an effective mutagenesis method using MNU treatment of fertilized egg cells at the single-cell stage immediately after fertilization. Chemical mutagen treatment of fertilized egg cells yields a higher mutation efficiency than dry seeds for two reasons: (1) M_1_ chimeric plants appear less frequently, and (2) selection of mutant cells due to competition between normal and mutant cells is reduced. The mutation efficiency of rice fertilized egg cells at the single-cell stage is twice as high as that of dry seeds (Satoh and Omura 1979).

Many genes responsible for morphological and physiological abnormalities have been identified using the MNU mutant libraries, demonstrating their usefulness in forward genetic approaches (Viana et al. 2019). Since chemical mutagens induce mutations randomly throughout the genome, a mutation screening step is required for each target gene. Targeting Induced Local Lesions In Genomes (TILLING) is one of the major screening methods to identify mutations in the targeted gene (Till et al. 2003). The TILLING method has identified numerous missense and nonsense mutations in genes that have not been previously identified in forward genetic screenings and have helped elucidate gene functions in plants (Kurowska et al. 2011). Thus, the MNU mutant libraries are useful as forward and reverse genetic tools in plant functional genomics.

As mentioned above, the TILLING method is an effective technology for obtaining mutants by reverse genetic screening; however, the conventional TILLING method requires a substantial amount of laboratory work to PCR screen mutants for each target gene (e.g., PCR evaluation of more than 2000–4000 individuals for nonsense or missense mutations). Recent next-generation sequencing (NGS) technology allows cost- and time-efficient determination of whole-genome sequences from multiple samples. NGS has been proposed to promote the mass identification of induced mutation sites and the construction of an in silico TILLING system as an online platform for screening mutations in target genes (Wang et al. 2012). Since rice has the smallest genome among cereal species and a high-quality reference genome, genome-wide sequencing of many individuals is becoming feasible. In this study, we sequenced a small MNU mutant library consisting of 266 M_1_ plants derived from the Japanese rice cultivar, Nipponbare, to examine the feasibility of an in silico TILLING system.

## Materials and Methods

### Plant Materials and Mutagenesis

The rice cultivar Nipponbare (*Oryza sativa* L. ssp. *japonica*) maintained at Kyushu University was used for mutagenesis. MNU mutagenesis of fertilized egg cells was conducted according to the method of Suzuki et al. (2008). Briefly, panicles with freshly pollinated flowers were dipped in a 1.0 mM MNU solution for 45 min at approximately 25 °C at 18 h after flowering. The M_1_ plants were grown and self-pollinated to obtain the leaf samples and M_2_ seeds in 2017–2019. The seed-setting frequency was measured from three panicles of each M_1_ plant.

### Whole-Genome Sequencing (WGS)

Total genomic DNA from the M_1_ plants was extracted from leaf samples frozen in liquid nitrogen using the CTAB method with some modifications (Murray and Thompson 1980). DNA libraries were constructed with a TruSeq Nano DNA Library Prep Kit (Illumina Co., Ltd.) and a NEBNext Ultra II FS DNA Library Prep Kit for Illumina (NEB). Pair-ends sequencing (2 × 150 bp) was conducted using an Illumina HiSeq X, HiSeq 2500, and NovaSeq 6000 (Illumina Co., Ltd.).

### Variant Discovery Analysis

Mutations were estimated according to recommendations in the Genome Analysis Tool Kit v 4.1.2.0 (GATK4) best practices (Poplin et al. 2018) with minor modification. Briefly, the obtained reads were qualified by Trimmomatic ver. 0.39 software (Bolger et al. 2014) using the parameter “ILLUMINACLIP:TruSeq3-PE.fa:2:30:10:2:keepBothReads LEADING:3 TRAILING:3 SLIDINGWINDOW:4:15 MINLEN:36” and mapped against Os-Nipponbare-Reference-IRGSP-1.0 pseudomolecules (Kawahara et al. 2013) using bwa-mem software (Li and Durbin 2010). Unaligned end sequences (soft clips) by the aligner were removed from the sam files with samclip software (https://github.com/tseemann/samclip) to suppress false positive variants found in the consequent analysis. After removing PCR duplicates with GATK4/Picard (http://broadinstitute.github.io/picard/), the apparent variants with quality scores of more than 100 were used to calibrate the.bam file with BaseQualityScore Recalibration in GATK4. The variant calls were identified by a haplotype caller and joint-genotyping in GATK4. Variant filtering was conducted by a hard filter suggested by GATK4 best practices. Indel variants in the Repeatmasked regions in the reference genome, obtained from gff files from The Rice Annotation Project database (RAP-DB), were removed by vcftools. All homozygous mutations were removed. For statistical analyses of the variants (.vcf file), vcftools ver. 0.1.15 (Danecek et al. 2011) and custom perl scripts were used. The effect of mutation on gene function was predicted using snpEff v4.1 with default parameters (Cingolani et al. 2012). For snpEff analysis, the gff files (IRGSP-1.0.51, 10-Mar-2021 and MSU release 7, 21-Nov-2011) were obtained from two rice genome annotation project sites (IRGSP-1.0: http://ftp.ensemblgenomes.org/pub/plants/release-51/gff3/oryza_sativa/, and MSU7: http://rice.plantbiology.msu.edu/pub/data/Eukaryotic_Projects/o_sativa/annotation_dbs/pseudomolecules/version_7.0/). The longest isoforms of the annotated genes were retrieved using snpEff analysis.

### Validation of the NGS Variants

Validation analyses of the NGS variants were accomplished by PCR and Sanger sequencing on an ABI3730xl DNA analyzer with BigDye Terminator v3.1. The same DNA samples used for the NGS analysis of the M_1_ plants were used for Sanger sequencing. Template DNAs containing the aimed mutations were amplified by PCR with KOD-Plus ver.2 polymerase (Toyobo, Osaka, Japan). Primers were designed based on the Nipponbare genome sequence using Primer3 (Untergasser et al. 2012) with default parameters. Primer sequences are listed in Additional file 1: Table S1. The PCR conditions were 35 cycles at 94 °C for 15 s, 60 °C for 20 s and 68 °C for 30 s. Amplicons were purified with NucleoSpin Gel and PCR Clean-up kits (Takara-Bio) and used for Sanger sequencing. An equal amount of leaf tissue from 8–12 M_2_ individual plants was collected to investigate the transmission frequency of mutations. DNA extracted from the bulked M_2_ samples was used for PCR and Sanger sequencing.

## Results

### Mutation Detection by Whole Genome Sequencing

**Fig. 1:**
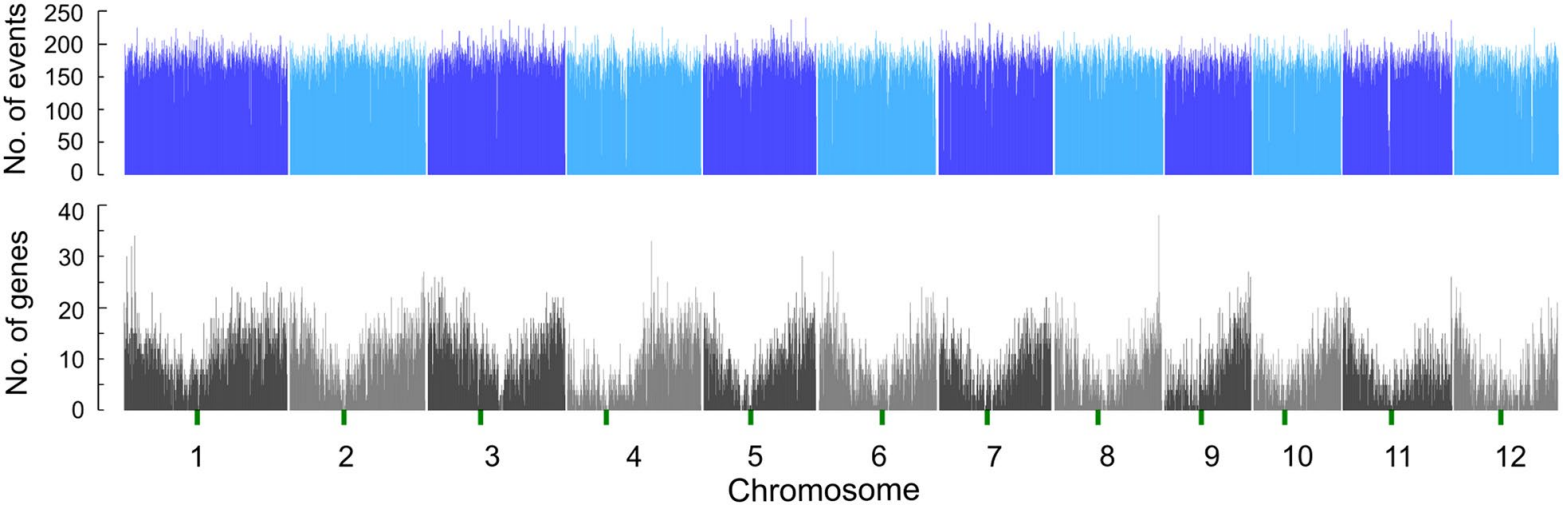
Distribution of SNVs along rice chromosomes at a 100 kb sliding window. The upper and lower bar charts represent the distributions of SNVs and genes annotated by IRGSP-1.0, respectively. The centromeres are marked with green vertical bars under each chromosome number in the gene distribution chart

In this study, we refer to MNU-induced single nucleotide substitutions and insertions/deletions as single nucleotide variants (SNVs) and InDels, respectively. To develop a mutant library for screening in silico mutants, cv. Nipponbare was mutagenized with MNU. Since Satoh et al. (2010) reported a weak negative correlation between the seed-setting percentage of M_1_ plants and the mutation rate, we selected 266 plants with a reduced ability to set seed from 4191 M_1_ individuals grown in 2017–2019. As a pilot study, we first sequenced the complete genomes of all 266 M_1_ plants by NGS analysis. The obtained short-read sequences (5.2–19.1 Gb for each individual, Average 8.5 Gb) were mapped onto the Nipponbare reference genome to identify small mutations such as SNVs and InDels. The average depth of coverage for each M_1_ individual genome was 14.9 times, and the average breadth of coverage of the reference genome was 95.3% (read depth ≧ 4) (Additional file 1: Table S2). Unique heterozygous variants present only in a single plant were defined as positive variants based on GATK joint genotyping for variant calls of multiple samples. Initially, 1,163,678 putative variants were detected [low-quality variants were cut off at a default setting quality value (QV) < 30 on the joint genotype vcf output] (Additional file 2: Fig. S1). To validate and determine reliable mutation sites in the M_1_ plants, 101 SNVs from eight individual M_1_ plants and their progeny (bulked M_2_ plant samples) were sequenced by Sanger sequencing (Additional file 2: Fig. S2). These SNVs were randomly selected from a broad range of QVs of SNVs having functional effects on IRGSP-1.0 genes, as described in the next paragraph “Characterization of SNVs”. Filtering at a threshold of QV > 80 was found to lead to a higher true positive percentage (98.7% in QVs > 80 threshold) (Additional file 2: Fig. S3, Additional file 1: Table S3). Therefore, we filtered out the lower quality SNVs (QVs > 80) and eventually obtained 656,669 SNVs from 266 M_1_ plants. The average number of SNVs per individual was 2468.7, ranging from 134 to 13,222, and the mutation rate was estimated to be 6.4 × 10^6^ per nucleotide (6.4 SNVs/Mb) (Additional file 1: Table S2). Of these SNVs, 91.2% were G/C to A/T transitions (Ts), whereas the A/T to G/C transitions and transversions (Tv) were less frequent (8.8%) (Table 1). The ratio of Ts/Tv was 14.81. The SNVs were equally distributed across the rice genome, both within and among chromosomes (Fig. 1, Additional file 1: Table S4). The vast majority of the variants detected here were SNVs and a few InDel mutations (0.47%, 3118 of 659,787 SNVs and InDels). The InDel mutations consisted of 632 insertions and 2486 deletions; 80.2% of the InDels were short insertions or deletions less than 3-bp in length (Additional file 2: Fig. S4). The false-positive percentage of InDel mutations was higher (31.3%, 5 of 16 InDels) than SNVs in the Sanger sequencing validation results (Additional file 1: Table S5).

**Table 1:** Classification of SNVs detected in the 266 M_1_ plants

Variant type*			
REF	ALT	No. of sites	%
A	C	1060	0.16
A	G	8000	1.22
A	T	11,768	1.79
C	A	7288	1.11
C	G	623	0.09
C	T	301,984	45.99
G	A	297,005	45.23
G	C	708	0.11
G	T	7336	1.12
T	A	11,776	1.79
T	C	8155	1.24
T	G	966	0.15
Total		656,669	100.00
Ts/Tv ratio		14.81 (615,144/41,525)	

*REF and ALT represent the reference base and the alternative base, respectively

### Functional Characterization of SNVs

**Fig. 2:**
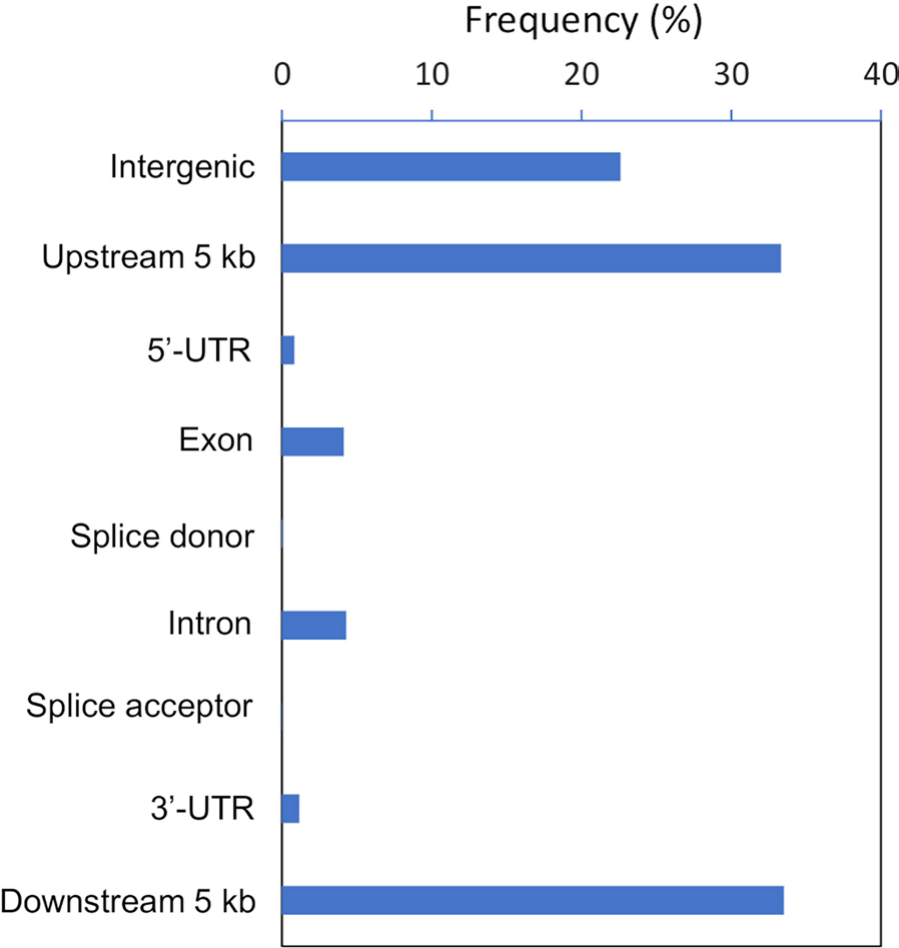
Annotation of SNVs classified based on their location in the IRGSP-1.0 genome

We annotated the functional effect of SNVs with the snpEff program. Most of the SNVs were located in intergenic regions (22.6%) or upstream/downstream regions (33.3/33.5%); 4.1% of SNVs were located in exons (Fig. 2). Of approximately 80 thousand SNVs found in the IRGSP-1.0 annotated gene database, 50,405 and 1753 SNVs led to missense and nonsense mutations, respectively (Table 2). More than 60% of all annotated genes we investigated (37,662 genes in IRGSP-1.0 and 55,718 genes in MSU7) had missense mutations, and approximately 5% had nonsense mutations. Of the 37,662 IRGSP-1.0 genes, 3248 genes had SNVs annotated as having high impact effects, including the loss of start and stop codons or splicing acceptor/donor sites (Additional file 1: Table S6). Therefore, approximately 70% of the annotated rice genes were covered by either nonsense or missense mutations in the 266 M_1_ mutants. In total, 17,052 genes had a synonymous variant that was classified as having a low-impact effect (Additional file 1: Table S6). We next investigated nucleobase bias in the upstream and downstream (± 20 bp) regions of all mutated guanine bases (G). A remarkable bias in nucleotide frequency was found at − 1 bp relative to G, where purine bases (A and G) were increased (15.8% for A and 18.7% for G) compared with randomly selected G bases (Additional file 2: Fig. S5). Moreover, a slight increase in T (6.4%) was found at + 1 bp. Other than at the − 1 and + 1 bp positions, there were no remarkable biases in the base composition within the 40 bp region we examined.

**Table 2:** Effect of SNVs on protein sequences

Type	IRGSP-1.0			MSU7		
	No. of SNVs	No. of genes	%*	No. of SNVs	No. of genes	%*
MISSENSE	50,405	23,081	61.3	99,436	38,775	69.6
NONSENSE	1753	1696	4.5	3966	3756	6.7
SILENT	28,360	17,093	45.4	52,674	28,864	51.8

*% Represents the proportion of genes containing one or more SNVs for each mutation type to the total annotated genes (IRGSP-1.0, 37,662 genes; MSU7, 55,718 genes)

### Phenotypic Effects of Mutations

**Fig. 3:**
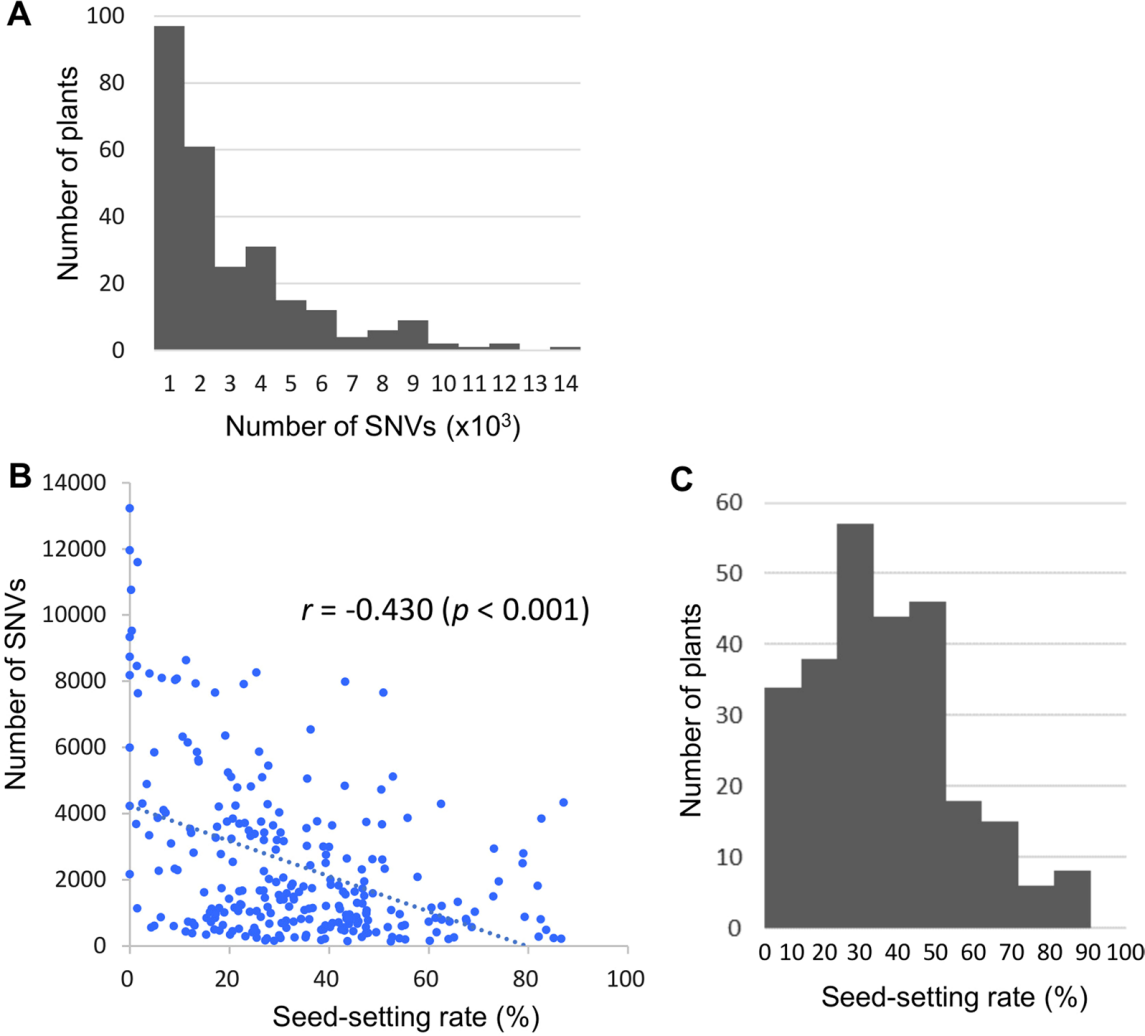
Correlation between the seed-setting percentage and the mutation frequency in the M_1_ population (*N* = 266). **A** Distribution of SNVs identified on 266 M_1_ genomes. **B** Correlation between the seed-setting percentage and the number of SNVs. **C** Distribution of the seed-setting percentage of the M_1_ mutants

To test the utility of our sequenced mutant library, we investigated the transmission frequencies of point mutations from M_1_ parents to M_2_ selfed progeny. For this purpose, DNA samples extracted from bulked M_2_ plants (*N* = 8–12) were sequenced by Sanger sequencing. These results were also used to validate the NGS variants as mentioned above. Of 92 true-positive SNVs, 78 (84.8%) were normally transmitted (Additional file 1: Table S7). For the InDels, 72.7% (8 of 11) were transmitted to the M_2_ progeny. We assume that some of the reduced transmission frequencies can be attributed to mutations affecting genes responsible for gamete development. Next, we investigated the relationship between the mutation rate and the phenotypic change frequency. The seed-setting percentages of the 266 M_1_ plants used for WGS analysis ranged from 0 to 87.1% (Average 34.7%). A weak negative correlation (*r* =  − 0.43, *p* < 0.001) was observed between the number of SNVs per individual and the seed-setting frequency of the M_1_ individuals (Fig. 3). In particular, M_1_ individuals with lower levels of seed settings (0–20% seed setting) tended to have a larger number of SNVs. This observation was consistent with results from a previous study in which the mutation rate was calculated by the occurrence of chlorophyll mutants in the M_2_ progeny (Satoh et al. 2010). To further test the phenotypic effects of the induced mutations found in the NGS analysis, small M_2_ populations (*N* = 15) derived from each M_1_ individual were grown, and their phenotypes were characterized. The M_2_ progeny exhibited a wide range of phenotypic variation in vegetative traits such as plant height, tiller number, leaf color, and reproductive traits such as spikelet shape and number of spikelets (Additional file 1: Table S8).

## Discussion

Conventional method for mutagenesis in plants includes treatment of dry seeds with a chemical mutagen. In recent years, there have been an increasing number of reports on genome-wide mutation analysis of EMS-mutants obtained using treated dry seeds (Sidhu et al. 2015; Jiao et al. 2016). However, there are no reports on the mutants obtained using fertilized egg cells treated with mutagens. In this study, we developed the Nipponbare mutant library using mutants induced by MNU treatment of the fertilized egg cells toward the construction of an in silico TILLING system. This is the first study to characterize features of genome-wide DNA mutations induced by MNU-treatment of plant fertilized egg cells. In our MNU-mutant library, approximately 656 K SNVs and 3.1 K small InDels were identified in 266 M_1_ plants. This result was equivalent to a mutation rate of one nucleotide change per 146 kb. This mutation rate was two-fold higher than that of other mutant libraries in diploid plant species (one nucleotide change in 294 kb in rice and 367 kb in tomato), indicating the high efficiency of mutation rate in our mutant library, and also suggesting our library as a prospective mutant resource for the construction of an in silico TILLING system. Our data showed that the most frequent changes were G/C to A/T transitions (91.2%). This finding is consistent with a previously proposed mechanism in which guanines are predominantly alkylated, and the guanines mismatched with thymine are replaced with adenines during DNA replication (Greene et al. 2003). Other mutation types (A/T to G/C transitions and transversions) were also found but were minor (8.8%). The results from previous studies and our study consistently indicate that chemical mutagenesis with an alkylating agent generates G/C to A/T transitions predominantly, although there are small variations among different plant species (e.g., 62% in wheat (Sidhu et al. 2015), 70% in tomato (Shirasawa et al. 2016), 96% in tobacco (Udagawa et al. 2021), 99% in Arabidopsis (Greene et al. 2003).

### Sequence Specificity of MNU Targets

We found that purine bases (A and G) were enriched at − 1 bp of mutated G nucleotides compared with randomly selected G nucleotides (Additional file 2: Fig. S5). A weak bias for nucleotide T was also found at + 1 bp. Studies in other plants have reported several different patterns of nucleotide frequency biases. A sorghum EMS mutant library had a high proportion of C nucleotides at − 2 bp and + 1 bp relative to the mutation site (Jiao et al. 2016). A TILLING study by Greene et al. (2003) reported an excess of purines at the ± 1 positions of mutated G nucleotides in Arabidopsis. This bias at the − 1 bp position was consistent with our result, but the bias for the + 1 bp position was different. We hypothesize that these differences in biases may be due to the distinctive treatment methods used to mutagenize different plant species.

### How can We Develop the Mutant Library Efficiently for In Silico TILLING?

The mutation frequency was weakly correlated with the seed-setting percentage of M_1_ individuals, a result consistent with a previous report by Satoh et al. (2010). Notably, M_1_ individuals with a lower seed-setting percentage (< 60%) tended to have a high mutation rate (some plants had over 7000 SNVs in Fig. 3). This observation suggested that selecting M_1_ individuals with a low seed-setting percentage (20–60%) was the most efficient way to develop the sequenced mutant library. Approximately half of the M_2_ lines in our mutant library showed remarkable morphological and physiological changes during growth, such as low germination, leaf color variation, and plant height. These phenotypic changes could be caused by any nonsense or missense mutation detected by WGS and can be used as good sources of germplasm for functional genomics investigations.

### Toward the Development of an In Silico TILLING System

Clustered regularly interspaced short palindromic repeats technology (CRISPR/Cas9), a method of gene editing, is a straightforward approach for obtaining target genes; however, gene editing techniques still need substantial laboratory work and tools, including at least two months to generate the mutants via Agrobacterium transformation for monocot species. In some countries, government regulations dictate that gene-edited plants must be treated in the same way as genetically modified organisms. Unlike gene targeting techniques, the whole-genome sequenced mutant library is ready for use. Researchers can start characterizing interesting mutants without heeding regulations, such as a fully closed greenhouse and the need to autoclave plant waste. We are developing an easy-to-use online screening system that allows the identification of mutations in the genes of interest by an in silico method. Mutant genome sequences will be available to the public for plant functional research through this online screening system. When this database is completed, the working time to find mutants of interest will be shortened from several weeks to several minutes. Additional WGS analysis of the residual M_1_ individuals is now being conducted (266 of 1384 mutants with reduced seed setting levels are reported in our study). Our results show that 266 M_1_ individuals account for 8.6% of the genes encoded by the rice genome as determined by high-impact mutations. This result implies that approximately 3000 M_1_ individuals would be sufficient to cover the entire rice genome. Therefore, a suitable population size for the mutant library would be more than 1500 individuals to cover half of all genes with high-impact SNVs and have 3.5 missense mutations per gene.

## Conclusions

In summary, we resequenced 266 rice mutants derived from MNU-treatment of fertilized egg cells and found 0.66 million induced point mutations. Over 60% of all annotated rice gene models harbored the nonsynonymous mutations in this mutant library. In the future, this proposed mutant library and its prospective database will allow rice researchers and other plant geneticists to study rice genes without requiring an immense amount of time and effort to make transgenic plants.

## Supplementary Information

**Additional file 1. Table S1.** Primer sets used for validation by PCR and Sanger sequencing. **Table S2.** NGS sequencing results of the 266 M_1_ mutants. **Table S3.** SNVs used for validation by Sanger sequencing. **Table S4.** Number of SNVs on each chromosome. **Table S5.** InDel variants used for validation by Sanger sequencing. **Table S6.** Classification of SNV effects predicted using snpEff. **Table S7.** Summary of the inheritance analysis of variants. **Table S8.** Abnormal phenotypes found in the M_2_ lines.

**Additional file 2. Fig. S1.** Distribution of quality values of SNVs detected in initial variant analysis (before removing low-quality value SNVs). A total of 1,163,678 SNVs were detected in the 266 M_1_ genomes. **Fig. S2.** Validation of NGS variants by Sanger sequencing. (**A**) Three mutation sites, NIM079-4, 143-2, and 047-10, found in three independent M_1_ plants based on NGS analysis are shown. (**B**) Sanger sequencing results of the NGS variants. The verified variant is marked with a black arrowhead. (**C**) Confirmation of variant inheritance from the M_1_ to the M_2_ generation. Bulked samples of the M_2_ progeny were sequenced. **Fig. S3.** Validation of the SNVs detected by NGS analysis. (**A**) A scatter plot showing the distribution of quality values for 101 SNVs derived from eight individual M_1_ plants that were used for the validation test. The X-axis indicates the plant ID for the eight individual M_1_ plants used in the validation analysis. The horizontal dotted line represents a quality value = 80. (**B**) Summary of the validation analysis. Percentage of true positives are shown with different thresholds of quality values (QV). **Fig. S4.** Size distribution of InDels found in the 266 M_1_ mutants. A total of 3118 InDels (2486 deletions and 632 insertions) were detected. **Fig. S5.** Nucleotide frequency at the flanking sequence (± 20 bp) of all mutated G nucleotides. (**A**) Nucleotide frequency around mutated G nucleotides (*n* = 305,049). (**B**) Nucleotide frequency around G nucleotides selected randomly for comparison (*n* = 305,049). Note: The average nucleotide content in Nipponbare whole genome is A: 28.2%, C: 21.8%, G: 21.8%, T: 28.2% (*n* = 373,245,519, based on the RAP-DB).

## Data Availability

The sequencing reads of the mutants in this paper have been deposited in the Sequence Read Archive of the DDBJ/GenBank data libraries under the Accession number DRA014011.

## Abbreviations

WGS: Whole-genome sequencing
NGS: Next-generation sequencing
MNU: N-methyl-N-nitrosourea
EMS: Ethyl methanesulfonate
TILLING: Targeting induced local lesions in genomes
SNV: Single nucleotide variant
InDel: Insertion/deletion

## References

[CR1] Bolger AM, Lohse M, Usadel B (2014). Trimmomatic: a flexible trimmer for Illumina sequence data. Bioinformatics.

[CR2] Cingolani P, Platts A, Wang LL, Coon M, Nguyen T, Wang L, Land SJ, Lu X, Ruden DM (2012) A program for annotating and predicting the effects of single nucleotide polymorphisms, SnpEff SNPs in the genome of Drosophila melanogaster strain w 1118 ; iso-2; iso-3. 10.4161/fly.19695 10.4161/fly.19695 PMC3679285 22728672

[CR28] Danecek P, Auton A, Abecasis G, Albers CA, Banks E, DePristo MA, Handsaker RE, Lunter G, Marth GT, Sherry ST, McVean G, Durbin R (2011). The variant call format and VCFtools. Bioinformatics (Oxford, England).

[CR3] Greene EA, Codomo CA, Taylor NE, Henikoff JG, Till BJ, Reynolds SH, Enns LC, Burtner C, Johnson JE, Odden AR, Comai L, Henikoff S (2003). Spectrum of chemically induced mutations from a large-scale reverse-genetic screen in Arabidopsis. Genetics.

[CR4] Jiao Y, Burke J, Chopra R, Burow G, Chen J, Wang B, Hayes C, Emendack Y, Ware D, Xin Z (2016). A sorghum mutant resource as an efficient platform for gene discovery in grasses. Plant Cell.

[CR5] Kawahara Y, de la Bastide M, Hamilton JP, Kanamori H, Mccombie WR, Ouyang S, Schwartz DC, Tanaka T, Wu J, Zhou S, Childs KL, Davidson RM, Lin H, Quesada-Ocampo L, Vaillancourt B, Sakai H, Lee SS, Kim J, Numa H, Matsumoto T (2013). Improvement of the *Oryza sativa* Nipponbare reference genome using next generation sequence and optical map data. Rice.

[CR6] Kurowska M, Daszkowska-Golec A, Gruszka D, Marzec M, Szurman M, Szarejko I, Maluszynski M (2011). TILLING—a shortcut in functional genomics. J Appl Genet.

[CR7] Li H, Durbin R (2010). Fast and accurate long-read alignment with Burrows–Wheeler transform. Bioinformatics.

[CR8] Muller HJ (1927). Artificial transmutation of the gene. Science.

[CR9] Murray MG, Thompson WF (1980). Rapid isolation of high molecular weight plant DNA. Nucleic Acids Res.

[CR10] Poplin R, Ruano-Rubio V, DePristo MA, Fennell TJ, Carneiro MO, Van der Auwera GA, Kling DE, Gauthier LD, Levy-Moonshine A, Roazen D, Shakir K, Thibault J, Chandran S, Whelan C, Lek M, Gabriel S, Daly MJ, Neale B, MacArthur DG, Banks E (2018). Scaling accurate genetic variant discovery to tens of thousands of samples. BioRxiv.

[CR11] Satoh H, Omura T (1979). Induction of mutation by the treatment of fertilized egg cell with N-methyl-IV-nitrosourea in rice. J Fac Agric Kyushu Univ.

[CR12] Satoh H, Matsusaka H, Kumamaru T (2010). Use of N-methyl-N-nitrosourea treatment of fertilized egg cells for saturation mutagenesis of rice. Breed Sci.

[CR13] Shirasawa K, Hirakawa H, Nunome T, Tabata S, Isobe S (2016). Genome-wide survey of artificial mutations induced by ethyl methanesulfonate and gamma rays in tomato. Plant Biotechnol J.

[CR14] Sidhu G, Mohan A, Zheng P, Dhaliwal AK, Main D, Gill KS (2015). Sequencing-based high throughput mutation detection in bread wheat. BMC Genom.

[CR15] Stadler LJ (1928). Mutations in barley induced by X-rays and radium. Science.

[CR16] Suzuki T, Eiguchi M, Kumamaru T, Satoh H, Matsusaka H, Moriguchi K, Nagato Y, Kurata N (2008). MNU-induced mutant pools and high performance TILLING enable finding of any gene mutation in rice. Mol Genet Genom.

[CR17] Till BJ, Reynolds SH, Greene EA, Codomo CA, Enns LC, Johnson JE, Burtler C, Odden AR, Young K, Taylor NE, Henikoff JG, Comai L, Henikoff S (2003). Large-scale discovery of induced point mutations with high-throughput TILLING. Genome Res.

[CR18] Udagawa H, Ichida H, Takeuchi T, Abe T, Takakura Y (2021). Highly efficient and comprehensive identification of ethyl methanesulfonate-induced mutations in *Nicotiana tabacum* L. by whole-genome and whole-exome sequencing. Front Plant Sci.

[CR19] Untergasser A, Cutcutache I, Koressaar T, Ye J, Faircloth BC, Remm M, Rozen SG (2012). Primer3—new capabilities and interfaces. Nucleic Acids Res.

[CR20] Viana VE, Pegoraro C, Busanello C, Costa de Oliveira A (2019). Mutagenesis in rice: the basis for breeding a new super plant. Front Plant Sci.

[CR21] Wang TL, Uauy C, Robson F, Till B (2012). TILLING in extremis. Plant Biotechnol J.

