# Supplementary material for: Whole-Genome Sequencing of Rice Mutant Library Members Induced by N-Methyl-N-Nitrosourea Mutagenesis of Fertilized Egg Cells
Source: Rice (N Y). 2022 Jul 16;15:38. doi: 10.1186/s12284-022-00585-1 (PMC9288566; doi:10.1186/s12284-022-00585-1)
Supplement: Supplementary file 2 — Additional file 2. Fig. S1. Distribution of quality values of SNVs detected in initial variant analysis (before removing low-quality value SNVs). A total of 1,163,678 SNVs were detected in the 266 M1 genomes. Fig. S2. Validation of NGS variants by Sanger sequencing. (A) Three mutation sites, NIM079-4, 143-2, and 047-10, found in three independent M1 plants based on NGS analysis are shown. (B) Sanger sequencing results of the NGS variants. The verified variant is marked with a black arrowhead. (C) Confirmation of variant inheritance from the M1 to the M2 generation. Bulked samples of the M2 progeny were sequenced. Fig. S3. Validation of the SNVs detected by NGS analysis. (A) A scatter plot showing the distribution of quality values for 101 SNVs derived from eight individual M1 plants that were used for the validation test. The X-axis indicates the plant ID for the eight individual M1 plants used in the validation analysis. The horizontal dotted line represents a quality value = 80. (B) Summary of the validation analysis. Percentage of true positives are shown with different thresholds of quality values (QV). Fig. S4. Size distribution of InDels found in the 266 M1 mutants. A total of 3118 InDels (2486 deletions and 632 insertions) were detected. Fig. S5. Nucleotide frequency at the flanking sequence (± 20 bp) of all mutated G nucleotides. (A) Nucleotide frequency around mutated G nucleotides (n = 305,049). (B) Nucleotide frequency around G nucleotides selected randomly for comparison (n = 305,049). Note: The average nucleotide content in Nipponbare whole genome is A: 28.2%, C: 21.8%, G: 21.8%, T: 28.2% (n = 373,245,519, based on the RAP-DB). [file 12284_2022_585_MOESM2_ESM.docx]

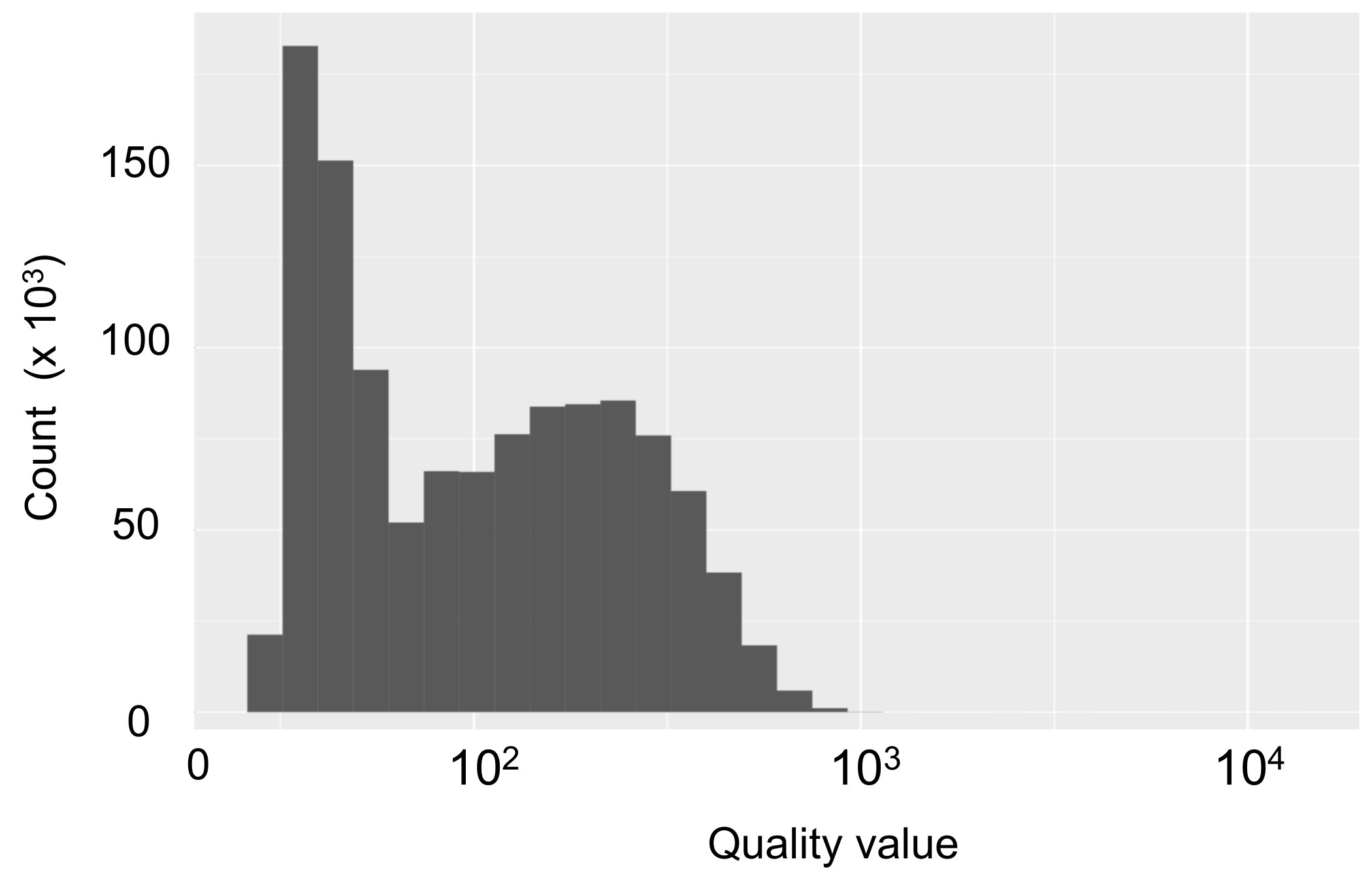


**Fig. S1.** Distribution of quality values of SNVs detected in initial variant analysis (before removing low-quality value SNVs). A total of 1,163,678 SNVs were detected in the 266 M_1_ genomes.


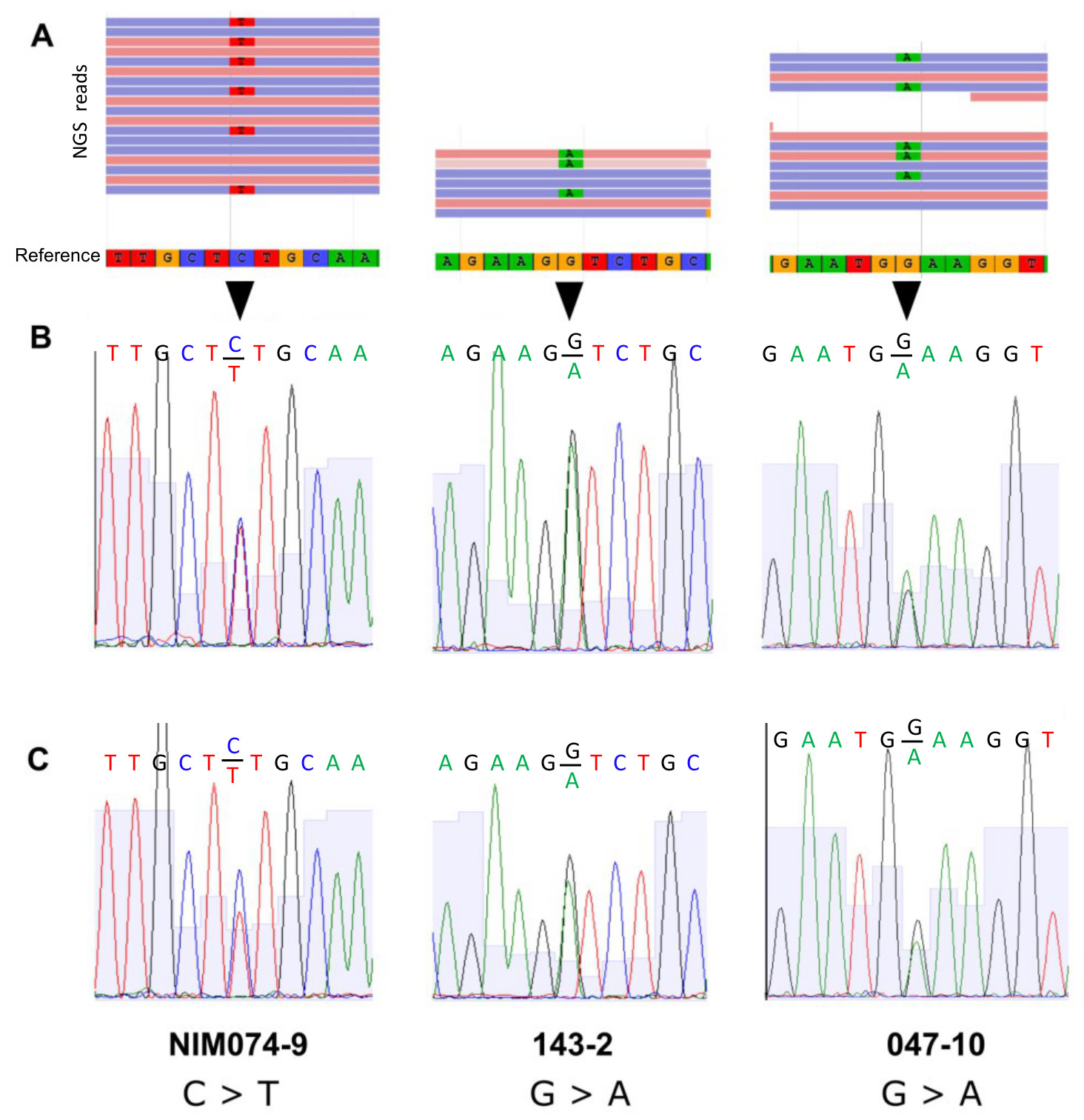


**Fig. S2.** Validation of NGS variants by Sanger sequencing. **(A)** Three mutation sites, NIM079-4, 143-2, and 047-10, found in three independent M_1_ plants based on NGS analysis are shown. **(B)** Sanger sequencing results of the NGS variants. The verified variant is marked with a black arrowhead. **(C)** Confirmation of variant inheritance from the M_1_ to the M_2_ generation. Bulked samples of the M_2_ progeny were sequenced.


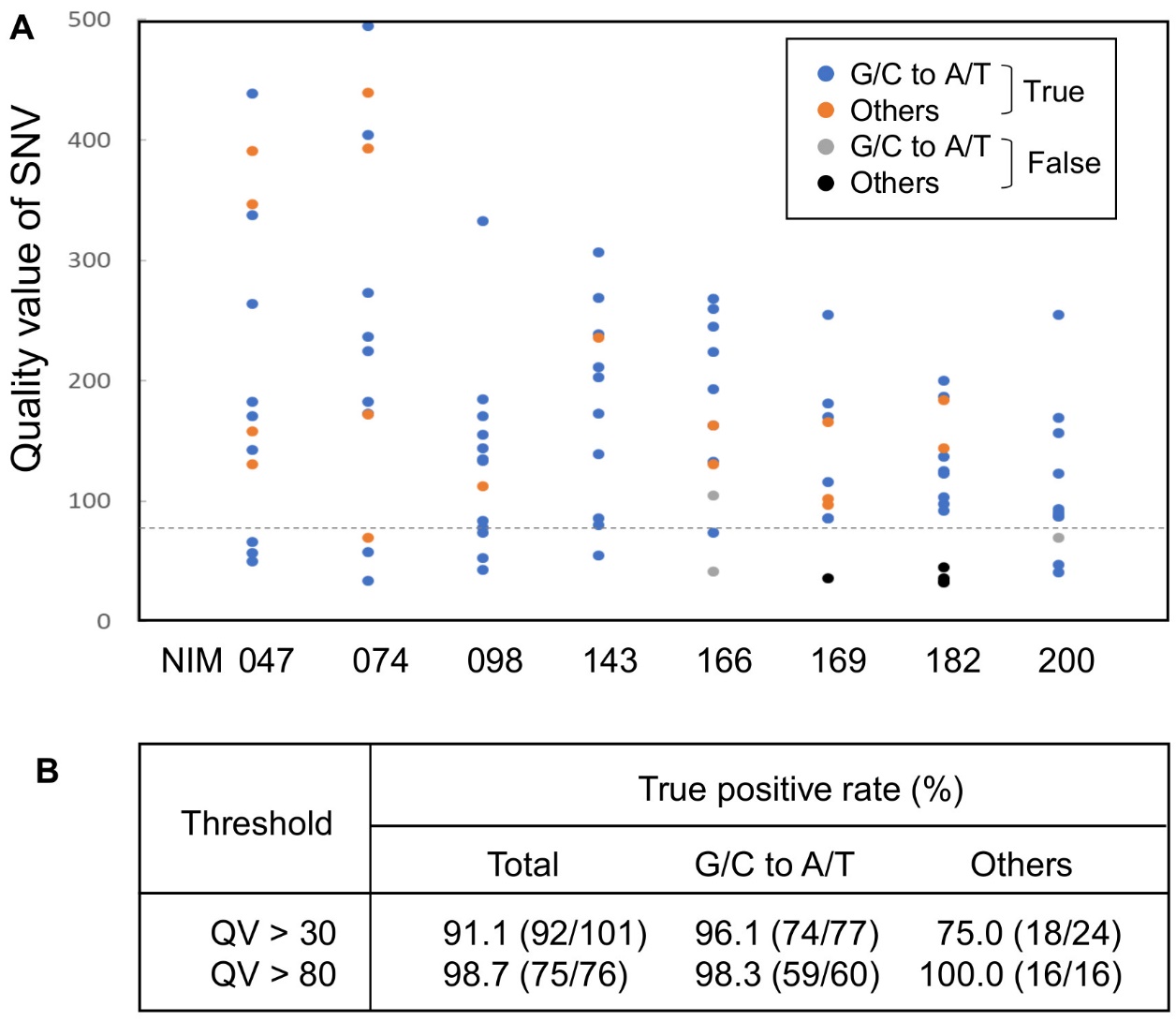


**Fig. S3.** Validation of the SNVs detected by NGS analysis. **(A)** A scatter plot showing the distribution of quality values for 101 SNVs derived from eight individual M1 plants that were used for the validation test. The X-axis indicates the plant ID for the eight individual M_1_ plants used in the validation analysis. The horizontal dotted line represents a quality value = 80. **(B)** Summary of the validation analysis. Percentage of true positives are shown with different thresholds of quality values (QV).


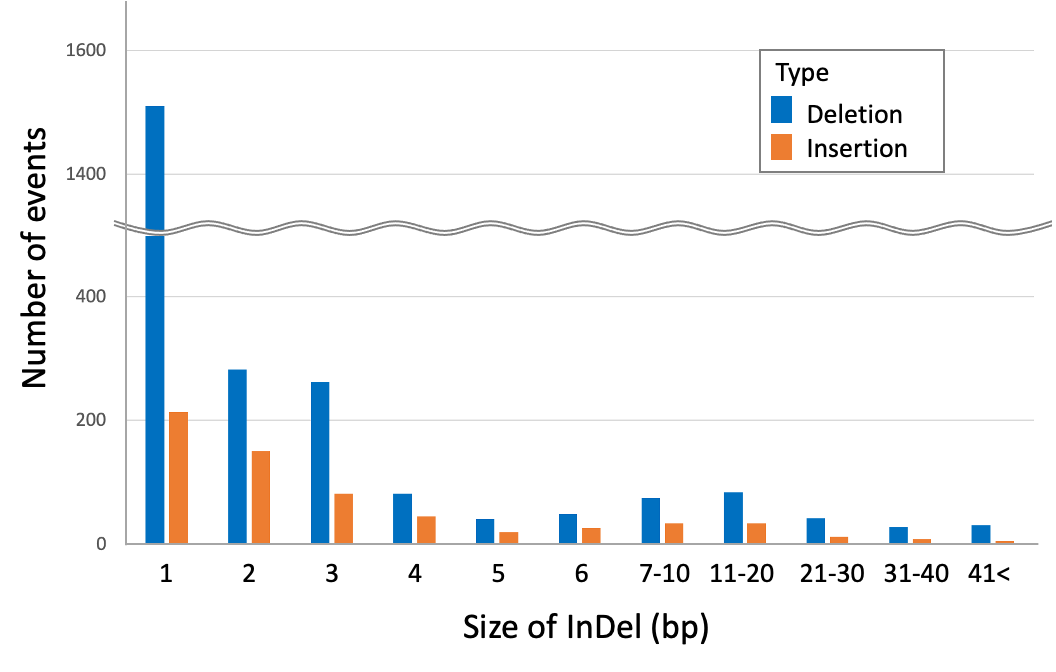


**Fig. S4.** Size distribution of InDels found in the 266 M_1_ mutants. A total of 3,118 InDels (2,486 deletions and 632 insertions) were detected.


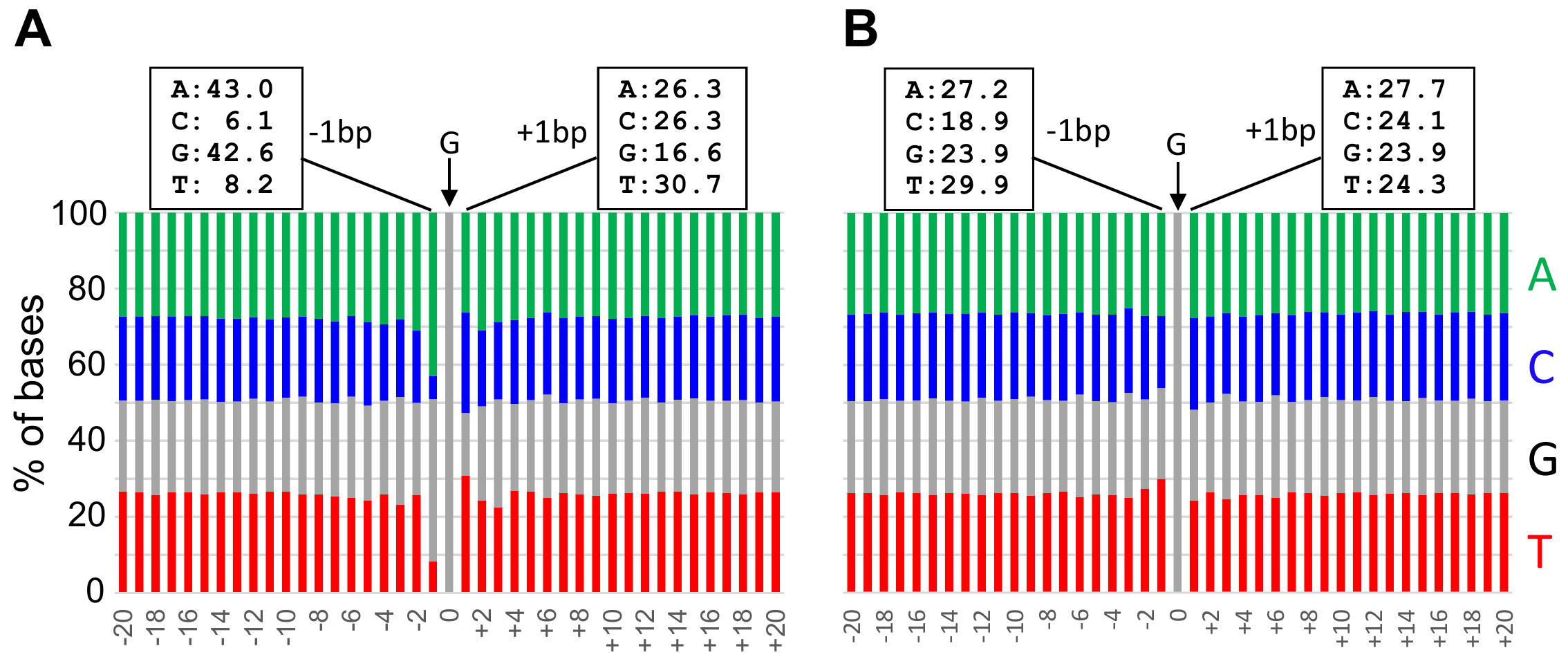


**Fig. S5.** Nucleotide frequency at the flanking sequence (±20 bp) of all mutated G nucleotides. **(A)** Nucleotide frequency around mutated G nucleotides (*n* = 305,049). **(B)** Nucleotide frequency around G nucleotides selected randomly for comparison (*n* = 305,049). Note: The average nucleotide content in Nipponbare whole genome is A: 28.2%, C: 21.8%, G: 21.8%, T: 28.2% (*n* = 373,245,519, based on the RAP-DB).
